# Metabolic Dysfunction Associated Fatty Liver Disease in Long-Term Cholecystectomy Patients: A Cross-Sectional Study

**DOI:** 10.5152/tjg.2024.24337

**Published:** 2024-11-25

**Authors:** Semih Sezer, Selim Demirci, Murat Kara

**Affiliations:** 1 Departments of Gastroenterology Medicine, Dr. Abdurrahman Yurtaslan Oncology Training and Research Hospital, Ankara, Türkiye; 2 Department of Physical and Rehabilitation Medicine, Hacettepe University Medical School, Ankara, Türkiye

**Keywords:** Cholecystectomy, metabolic-associated fatty liver disease, ultrasound, hepatosteatosis

## Abstract

**Background/Aims:**

Cholecystectomy, while generally safe with low perioperative morbidity and mortality, has been linked to an increase in metabolic disorders. Metabolic dysfunction-associated fatty liver disease (MAFLD) is a globally prevalent condition that leads to both hepatic and systemic complications. This study aimed to investigate the association between cholecystectomy and MAFLD.

**Materials and Methods:**

This cross-sectional study was designed to evaluate the relationship between cholecystectomy and MAFLD. Metabolic dysfunction-associated fatty liver disease was defined by the presence of hepatic steatosis in combination with any of the following conditions: diabetes mellitus (fasting plasma glucose ≥126 mg/dL), overweight (body mass index (BMI) ≥25 kg/m^2^), or metabolic dysregulation.

**Results:**

A total of 163 participants with BMI ≥25 kg/m^2^, including consecutive cholecystectomized (N = 83) and non-cholecystectomized (N = 80) subjects, were included. The prevalence of MAFLD was found in 64 out of 83 (77.1%) cholecystectomized patients and in 30 out of 80 (37.5%) non-cholecystectomized subjects (*P* < .001). When age, gender, BMI, exercise habits, hypertension, diabetes mellitus, and cholecystectomy status were included in regression analyses, we found that only BMI [odds ratio (OR) = 1.155 (95% CI: 1.040-1.283)] and cholecystectomy [OR = 4.540 (95% CI: 2.200-9.370)] were independently associated with MAFLD (both *P* < .01). ROC analysis identified 10 years as the cut-off, with MAFLD risk being 2.7-7.3 times higher in patients with cholecystectomy for ≤10 and >10 years.

**Conclusion:**

In our study, MAFLD was found to be 4.5 times more likely in cholecystectomized patients compared to those without cholecystectomy, with a significant increase in frequency observed after 10 years. These results suggest that cholecystectomized patients should be monitored for MAFLD.

**Main Points**

- MAFLD has been observed with an odds ratio of 4.5 in cholecystectomized patients compared to those without.
- MAFLD frequency notably increases 10 years after cholecystectomy.
- Cholecystectomy patients should be warned about the long-term risk of MAFLD.

## Introduction

Cholecystectomy is a surgical procedure that is widely performed globally, exhibiting a low rate of morbidity and mortality.^1^ Cholecystectomy is considered a relatively harmless procedure with no harmful effects.^2^ However, the unexplained increase in metabolic disorders such as dyslipidemia, hyperglycemia, non-alcoholic fatty liver disease (NAFLD), and related complications with cholecystectomy has led to the need for further investigation of cholecystectomy patients.^3-5^

Non-alcoholic fatty liver disease is a worldwide epidemic with an average prevalence of 25%.^6^ The prevalence of metabolic dysfunction-related fatty liver disease (MAFLD) in the Turkish population was found to be 45%.^7^ It can present with a wide range of clinical manifestations, from simple fatty liver disease to hepatocellular carcinoma.^8^ Mortality due to extrahepatic causes is also high in patients with NAFLD.^9^ Recently, it has been recognized that it would be more accurate to call it MAFLD rather than NAFLD.^10^ The diagnostic criteria for MAFLD include histologic or imaging detection of hepatic steatosis with metabolic disorder factors as a prerequisite for making a diagnosis.^10^

The aim of this study was to examine the association of cholecystectomy with MAFLD in the long term.

## Materials and Methods

This cross-sectional study was planned to evaluate the relationship between patients who had undergone cholecystectomy and MAFLD. The study encompassed individuals aged 18 and older with a body mass index (BMI) ≥25 kg/m^2^ who visited the Dr. Abdurrahman Yurtaslan Oncology Training and Research Hospital gastroenterology clinics between November 2023 and August 2024. Exclusion criteria for the cholecystectomy group included having undergone cholecystectomy less than 5 years prior, pregnancy or breastfeeding, factors that may lead to inaccuracies in the assessment of steatosis on ultrasound (US) (e.g., advanced chronic kidney disease, liver cirrhosis), and any urgent gastroenterological conditions. The control group was selected from individuals who had not undergone cholecystectomy or any biliary tract surgery, using similar exclusion criteria as those applied to the cholecystectomy group. Control group participants were sequentially randomized and selected to match the cholecystectomy group in terms of age and gender until the target sample size was reached. The 2 groups (with and without cholecystectomy) were compared in terms of MAFLD. Clinical data, including age, BMI, education level, exercise habits, current smoking, and comorbidities (e.g., hypertension, diabetes mellitus (DM), and hyperlipidemia) were collected. Participants exercised habits if they did moderate intensity of physical activity (>30 min/day) at least 3-4 days in a week. Participants were provided with details about the study and gave their written informed consent. The study received approval from the Dr. Abdurrahman Yurtaslan Oncology Training and Research Hospital Ethics Committee (date: November 1, 2023, Decision no. 2023-10/417). ClinicalTrials registration number was obtained as NCT06443723.

### Anthropometric and Laboratory Measurements

Height (cm) and weight (kg) were measured using standardized protocols. The midpoint between the iliac process and the lower rib border was used to estimate waist circumference (WC). After a minimum of 8 hours of fasting, samples of blood were taken from a peripheral vein, and values were noted for hemoglobin, glucose, low-density lipoprotein (LDL), triglycerides (TG), high-density lipoprotein (HDL), thyroid-stimulating hormone, alanine aminotransferase (ALT), aspartate aminotransferase, and gamma-glutamyl transferase (GGT).

### Ultrasonographic Assessment

**Figure 1.**
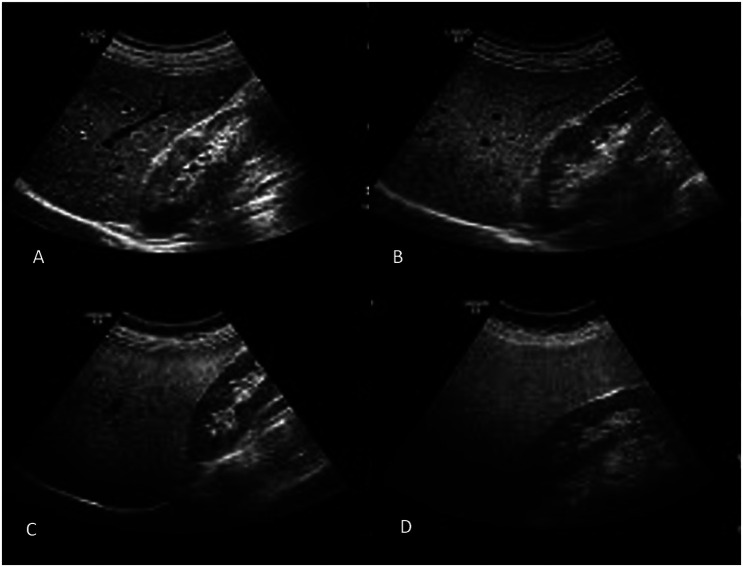
Ultrasonographic grading of hepatosteatosis.

Hepatobiliary US was performed by an experienced physician using a 7-12 MHz linear probe (Mindray DC-7, China). Classification was made using features such as parenchymal brightness, the liver and kidney’s contrast, the appearance of intrahepatic arteries, and the diaphragm. Steatosis was classified into 4 grades based on liver fat accumulation and echogenicity. Grade 0 (normal) indicates no fat accumulation, with normal parenchymal echogenicity. Grade 1 (mild steatosis) involves mild fat accumulation where liver echogenicity is slightly increased but the contrast between the liver and the diaphragm or kidneys remains intact. Grade 2 (moderate steatosis) shows moderate fat accumulation with a significant increase in liver echogenicity, making it difficult to visualize the diaphragm or kidneys. Finally, grade 3 (severe steatosis) is characterized by severe fat accumulation, with markedly increased liver echogenicity, rendering the diaphragm or kidneys nearly invisible on US (Figure 1).^11,12^

### Metabolic Dysfunction-Associated Fatty Liver Disease Diagnostic Criteria

The presence of hepatic steatosis in combination with any of the following: DM (fasting plasma glucose ≥126 mg/dL), overweight; BMI ≥25 kg/m^2^ or metabolic dysregulation was defined as MAFLD. Metabolic dysregulation refers to a lack of coordination and imbalances in a number of metabolic pathways. Metabolic dysregulation was defined as ≥2 metabolic abnormalities (WC ≥102 cm in men, ≥88 cm in women, blood pressure ≥130/85 mm Hg, TG ≥150 mg/dL or antihypertensive therapy intake, HDL <40/50 mg/dL (male/female) or antilipid therapy intake, prediabetes (i.e., fasting glucose levels between 100 and 125 mg/dL, or 2-hour post-load glucose levels 140-199 mg/dL or HbA1c 5.7%-6.4%), homeostatic model assessment score (HOMA) ≥2.5, high-sensitivity C-reactive protein (hs-CRP) levels >2 mg/dl.^13^

### Calculation of Fatty Liver Index

We used the fatty liver index (FLI), one of the non-invasive indicators for diagnosing fatty liver disease (FLD) in patients without significant alcohol consumption. The FLI was calculated using measurements of BMI, WC, TG, and GGT. The following formula was used to produce a score ranging from 1 to 100:

FLI = (e^0.953*log e(triglycerides) + 0.139*BMI + 0.718*log e(GGT) + 0.053*waist circumference − 15.745^)/(1 + e^0.953*log e(triglycerides) + 0.139*BMI + 0.718*log e(GGT) + 0.053*WC − 15.745^) × 100

We used a value of <30 (negative likelihood ratio = 0.2) to indicate the absence of FLD, and a value of ≥60 (positive likelihood ratio = 4.3) to indicate the presence of FLD.^14^

### Statistical Analysis

SPSS version 23.0 (IBM SPSS Corp.; Armonk, NY, USA) was used. Numbers and percentages (%) are used to represent categorical variables, and the mean ± SD is used to represent numerical variables. The normality of the distribution was assessed using the Kolmogorov–Smirnov test. Comparisons between groups were performed using Student’s *t* or Mann–Whitney *U*-test, depending on the normality, for numerical variables, and using the chi-square or Fisher’s exact test for categorical variables.

For the multivariate analysis, the possible factors (i.e., age, gender, BMI, smoking, exercise, hypertension, DM, and cholecystectomy) were further entered into the binary logistic regression analysis (with the enter method) to determine independent predictors for predicting MAFLD. Since there was an independent relationship between MAFLD and duration of cholecystectomy in the cholecystectomized subgroup, absolute and cut-off values (according to receiver operating characteristic analysis) for duration of cholecystectomy were used in separate regression models. The Hosmer–Lemeshow test was used to see whether the regression analysis suited the data. *P* < .05 was used to determine statistical significance.

## Results

**Figure 2.**
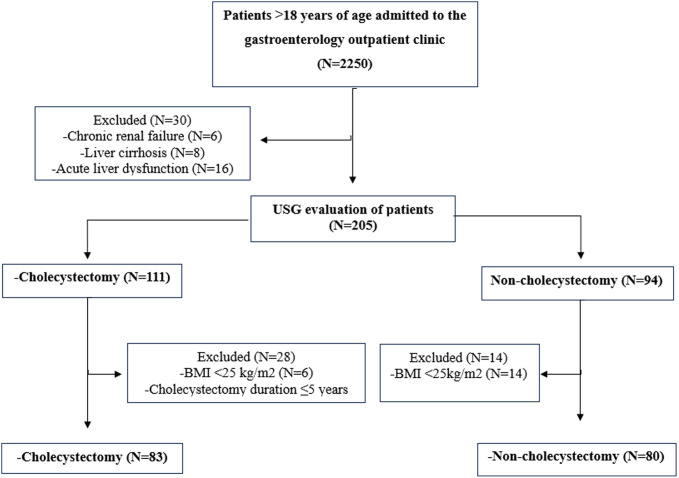
Flowchart of the study.

A total of 163 patients with a BMI ≥25 kg/m^2^, comprising 83 who had undergone cholecystectomy and 80 who had not, were included in the study (Figure 2). The age range of the participants was between 25 and 84 years. For those who had undergone cholecystectomy, the mean duration since the procedure was 14.0 ± 7.9 years, with the shortest duration being 5 years and the longest being 50 years. While 78 of 83 cholecystectomy patients (94.0%) were operated on due to cholelithiasis, the remaining 5 patients (6.0%) were operated on for other reasons. Due to missing data, the FLI score could not be calculated for 20 patients.

The comparison of clinical characteristics of patients with MAFLD (N = 94) and without MAFLD (N = 69) is given in Table 1. In participants with a BMI ≥ 25 kg/m^2^, the prevalence of MAFLD was observed in 64 out of 83 cholecystectomized patients (77.1%) compared to 30 out of 80 non-cholecystectomized individuals (37.5%), with statistical significance (*P* < .001). When compared to patients without MAFLD, BMI (31.4 ± 3.8 kg/m^2^ vs. 29.0 ± 3.5 kg/m^2^), frequency of cholecystectomy (68% vs. 27.5%), duration of cholecystectomy (14.8 ± 8.2 years vs. 11.4 ± 6.5 years), and presence of FLD (72.8% vs. 38.7%) were higher, while doing exercise (25.5% vs. 40.6%) was lower in patients with MAFLD (all *P* < .05). Among laboratory parameters, glucose, ALT, GGT, and TG levels were higher in patients with MAFLD compared to those without MAFLD (all *P* < .05).

Of the patients evaluated, 69 were classified as Grade 0 on US. Among those diagnosed with MAFLD, 48 patients (51.1%) were observed to have Grade 1, 34 patients (36.2%) had Grade 2, and 12 patients (12.8%) had Grade 3 steatosis.

When binary logistic regression analysis was used to assess the association among age, gender, BMI, smoking, exercise, hypertension, DM, and cholecystectomy (Table 2), only BMI [odds ration (OR) = 1.155 (95% CI: 1.040-1.283)] and presence of cholecystectomy [OR = 4.540 (95% CI: 2.200-9.370)] were independently associated with the presence of MAFLD (both *P* < .01).

When the same parameters were analyzed to predict MAFLD in the cholecystectomized group (N = 83), only BMI [OR = 1.253 (95% CI: 1.012-1.552)] and duration of cholecystectomy [OR = 1.131 (95% CI: 1.016-1.259)] were positively and independently significant (both *P* < .05). According to ROC analysis, 10 years was identified as the cut-off value, with an area under the curve of 0.649 (95% CI: 0.503-0.795). When binary regression analysis was repeated for this cut-off value (10 years), MAFLD was found to be 2.697 times more prevalent (95% CI: 1.096-6.634) in patients who had undergone cholecystectomy ≤10 years ago and 7.272 times more prevalent (95% CI: 2.853-18.540) in those who had undergone cholecystectomy >10 years ago, compared to the group without cholecystectomy (both *P* < .001).

## Discussion

In our study, among patients with a BMI ≥25 kg/m^2^, MAFLD was highly prevalent (77.1%) in cholecystectomized individuals compared to non-cholecystectomized subjects (37.5%). In addition, independent of confounding factors, MAFLD was observed with an odds ratio of 4.5 in cholecystectomized patients compared to those without, with its frequency notably increasing after 10 years.

Cholecystectomy is an effective treatment method with low mortality and morbidity, employed to prevent certain life-threatening health issues (acute or chronic cholecystitis, cholangitis, and biliary pancreatitis) and long-term health risks associated with gallbladder pathology (malignancy).^15,16^ In contrast, cholecystectomy may lead to some metabolic problems in the long term by affecting digestive physiopathology.

The gallbladder’s role in regulating postprandial bile acid release is crucial for lipid metabolism and glucose balance. After a cholecystectomy, continuous bile acid flow into the small intestine can impact significant metabolic pathways, notably fibroblast growth factor 19 (FGF19) and the protein-coupled bile acid receptor (TGR5).^17^ FGF19, a hormone produced by the small intestine, normally inhibits bile acid production in the liver. Post-cholecystectomy, reduced levels of FGF19 may lead to increased hepatic bile acid synthesis, potentially causing liver fat accumulation.^17-19^ Additionally, bile acids stimulate the release of glucagon-like peptide-1 through TGR5 receptors, which improves insulin sensitivity. Reduced TGR5 activity following cholecystectomy might result in hepatic insulin resistance and an increased risk of NAFLD.^20,21^ Changes in the gut microbiota post-surgery could also heighten intestinal permeability, allowing endotoxins to enter the portal circulation and induce liver inflammation.^22^

In a meta-analysis, Ayada et al^23^ found that the prevalence of MAFLD was 33%, while the prevalence of NAFLD was 29.1% in the general population. The prevalence of MAFLD in the Turkish population is 45%.^7^ Few research have been conducted to date on the relationship between NAFLD and cholecystectomy in various ethnic groups. Large population-based studies have shown that among those who have undergone cholecystectomy, the prevalence of NAFLD (48.4%) is 2.4 times higher in the United States population and 1.4 times higher in the Asian population.^4,24^ In a meta-analysis of 20 studies, this rate has been identified to be about 1.5 times higher.^25^ In our study, we found that, independent of confounding factors, MAFLD was about 4.5 times more prevalent in patients who had undergone cholecystectomy compared to those who had not. The prevalence of MAFLD among participants without cholecystectomy was similar to the prevalence reported by Ayada et al in their meta-analysis and by Yılmaz et al in the Turkish population. The higher prevalence of MAFLD among cholecystectomized individuals compared to United States and Asian populations may be related to genetic and dietary habits specific to the population, the inclusion of participants with a BMI ≥25 kg/m^2^, and the use of inclusion criteria for MAFLD, which differ from the exclusion criteria used for NAFLD. Ruhl and Everhart^4^ found a significant association between cholecystectomy and liver enzyme elevation in patients more than 10 years postoperatively. In our study, similar to the findings of Ruhl and Everhart, we observed a significant association between cholecystectomy and the development of MAFLD in participants who had undergone cholecystectomy more than 10 years prior. In the MAFLD group, glucose, ALT, GGT, and TG levels were significantly elevated compared to the non-MAFLD group.

This research has some advantages over previous research. First, since US evaluations and demographic examinations of patients were performed by a single experienced physician in this study, operator-induced standard differences were minimized. Second, it is the first study to use recent diagnostic criteria for MAFLD in cholecystectomy patients. Third, in our population, this investigation validated the independent correlation between cholecystectomy and MAFLD.

The limitations of this study are as follows: First, we diagnosed MAFLD by US instead of histologic examination. However, the histologic diagnosis of MAFLD is difficult to perform in the general population. Our finding of a significant difference in MAFLD in patients with FLI < 30 and FLI > 60 confirmed our US scan evaluation. Second, we did not routinely assess HOMA and hs-CRP in these patients. However, this deficiency did not affect our results because other criteria for metabolic syndrome were met. Finally, we did not have any data about the MAFLD status of patients before undergoing a cholecystectomy procedure. However, binary logistic regression analysis revealed that cholecystectomy exhibited a stronger association with MAFLD compared to other established risk factors.

In conclusion, this study has demonstrated a 4.5-fold increased odds ratio for MAFLD in patients who have undergone cholecystectomy compared to those who have not. This relationship was found to intensify with the duration of time since the cholecystectomy, particularly after 10 years. In this context, it can be recommended that patients be evaluated for MAFLD in the long term following cholecystectomy and that appropriate preventive measures be considered.

## Figures and Tables

**Table 1.** Demographic and Clinical Characteristics of the Participants (N = 163)

**Characteristic**	**Controls **(N = 69)	**MAFLD **(N = 94)	***P***
Age (year)	60.5 ± 8.8	60.0 ± 10.7	.791
Gender, male	20 (29.0)	23 (24.5)	.518
BMI (kg/m^2^)	29.0 ± 3.5	31.4 ± 3.8	**<.001**
Waist circumference (cm)	101.0 ± 10.2	104.4 ± 11.0	.050
Smoking Alcohol, social drinking	12 (17.4) 1 (1.4)	26 (27.7) 7 (7.4)	.126 .140
Education (year)	5 (5-5)	5 (5-8)	.583
Exercise	28 (40.6)	24 (25.5)	**.042**
Comorbidities			
Hypertension	27 (39.1)	50 (53.2)	.076
DM	16 (23.2)	34 (36.2)	.076
Hyperlipidemia	5 (7.2)	9 (9.7)	.586
Cholecystectomy	19 (27.5)	64 (68.1)	**<.001**
Faty liver index			**<.001**
<30	10 (16.1)	5 (6.2)	
30-59	28 (45.2)	17 (21.0)	
≥60	24 (38.7)	59 (72.8)	
Laboratory test			
Hemoglobin (g/dL)	13.6 ± 1.5	13.7 ± 1.5	.865
Glucose (mg/dL)	98.1 ± 21.7	117.1 ± 40.7	**<.001**
ALT (U/L)	18.7 ± 8.9	23.0 ± 13.1	**.017**
AST (U/L)	19.0 ± 5.2	21.2 ± 12.4	.652
GGT (U/L)	21.7 ± 15.4	29.6 ± 16.4	**<.001**
Triglyceride (mg/dL)	128.0 ± 71.9	155.5 ± 71.7	**.023**
LDL (mg/dL)	119.8 ± 32.0	126.3 ± 34.6	.273
HDL (mg/dL)	54.5 ± 12.8	52.3 ± 12.4	.278
TSH (mIU/L)	2.06 ± 1.38	2.10 ± 1.46	.911

Results are presented as mean ± SD, median (25%-75% inter-quartiles), and number (%). Statistically significant values are shown in bold.

ALT, alanine aminotransferase; AST, aspartate aminotransferase; BMI, body mass index; DM, diabetes mellitus; GGT, gamma-glutamyl transferase; HDL, high-density lipoprotein; LDL, low-density lipoprotein; MAFLD, metabolic associated fatty liver disease; TSH, thyroid stimulating hormone.

**Table 2.** Binary Logistic Regression Analysis Predicting MAFLD (N = 163)

Parameter	OR	95% CI	*P*
Age	0.992	0.956-1.030	.675
Gender, male	1.145	0.482-2.723	.759
BMI	1.155	1.040-1.283	**.007**
Smoking	1.349	0.556-3.275	.509
Exercise	0.640	0.283-1.445	.283
Hypertension	1.199	0.541-2.655	.655
DM	1.494	0.666-3.352	.331
Cholecystectomy	4.540	2.200-9.370	**<.001**

Statistically significant values are shown in bold.

BMI, body mass index; DM, diabetes mellitus; MAFLD, metabolic associated fatty liver disease; OR, odds ratio.

## Data Availability

The data that support the findings of this study are available on request from the corresponding author.
